# Disclosure of Same-Sex Sexual Practices to Family and Healthcare Providers by Men Who Have Sex with Men and Transgender Women in Nigeria

**DOI:** 10.1007/s10508-020-01644-8

**Published:** 2020-03-19

**Authors:** Afoke Kokogho, Senate Amusu, Stefan D. Baral, Manhattan E. Charurat, Sylvia Adebajo, Olumide Makanjuola, Veronica Tonwe, Casey Storme, Nelson L. Michael, Merlin L. Robb, Julie A. Ake, Rebecca G. Nowak, Trevor A. Crowell, for the TRUST/RV368 Study Group

**Affiliations:** 1 U.S. Army Medical Research Directorate–Africa, Nairobi, Kenya; 2 HJF Medical Research International, Abuja, Nigeria; 3 Johns Hopkins Bloomberg School of Public Health, Baltimore, MD USA; 4 Institute of Human Virology, University of Maryland, Baltimore, MD USA; 5 Population Council Nigeria, Abuja, Nigeria; 6 The Initiative for Equal Rights, Lagos, Nigeria; 7 Henry M. Jackson Foundation for the Advancement of Military Medicine, Bethesda, MD 20817 USA; 8 U.S. Military HIV Research Program, Walter Reed Army Institute of Research, Silver Spring, MD USA; 9 Center for Infectious Disease Research, Walter Reed Army Institute of Research, Silver Spring, MD USA

**Keywords:** Gender and sexual minorities, AIDS, Nigeria, Sexual orientation, Stigma, Transgender

## Abstract

Disclosure of same-sex sexual practices by men who have sex with men (MSM) and transgender women (TGW) may facilitate appropriate healthcare engagement, including risk assessment for HIV and other sexually transmitted infections (STIs), and negotiation of condom use with partners. However, disclosure may also generate stigma. In these cross-sectional analyses, MSM and TGW were categorized based on self-report of disclosure to family members and healthcare providers (HCP) at enrollment into the TRUST/RV368 study of comprehensive HIV and STI care programs in Abuja and Lagos, Nigeria. Multivariable Poisson regression models with robust error variance were used to estimate relative risk of disclosure with 95% confidence intervals. Pearson’s chi-squared test was used to compare condom use and stigma indicators by disclosure status. Of 2557 participants who answered baseline questions about disclosure, 384 (15.0%) had ever disclosed to a family member and 733 (28.7%) to HCP, including 192 (7.5%) who disclosed to both. Higher education, prevalent HIV infections, and residence in Lagos were each associated with increased likelihood of disclosure to family and HCP. Older participants were more likely to disclose to HCP but not family. Participants who made a disclosure to family or HCP were more likely to report condom use during anal sex as well as perceived and experienced stigma that included healthcare avoidance, blackmail, assault, and sexual violence as compared to participants who had not disclosed. Improved disclosure practices within safe spaces may enhance engagement of MSM and TGW in healthcare and HIV prevention services.

**Electronic supplementary material:**

The online version of this article (10.1007/s10508-020-01644-8) contains supplementary material, which is available to authorized users.

## Introduction

For men who have sex with men (MSM) and transgender women (TGW), disclosure of same-sex sexual practices can be a critical step toward engaging healthcare services to appropriately prevent and treat HIV and other sexually transmitted infections (STIs) (Metheny & Stephenson, 2016). Disclosure of a concealable and stigmatized sexual identity may lead to improved healthcare outcomes by allowing MSM and TGW to express personally relevant information, garner social support, and influence societal views (Chaudoir & Fisher, 2010). Disclosure to family members can lead to acceptance, support, and identity formation that can lead to positive psychological adjustments and, in turn, may promote adherence to safer sex practices and medication, including pre-exposure prophylaxis (PrEP) or antiretroviral therapy (ART) for HIV (Elizur & Ziv, 2001). Disclosure to healthcare providers (HCPs) is necessary to receive anatomically appropriate screening for STIs and to inform the frequency of such screening.

While same-sex sexual practices have become culturally more acceptable in many Western countries—with associated positive gains in human rights for MSM and TGW—the same has not been observed in many parts of the developing world, including sub-Saharan Africa, where there can be dire consequences for being identified as homosexual, including stigmatization, assault, imprisonment, and even capital punishment (Bailey et al., 2016). Therefore, disclosure of same-sex sexual practices often occurs at substantial risk to the person making the disclosure (Mason et al., 2015; Risher et al., 2013; Schrimshaw, Downing, & Cohn, 2018; Serovich, Grafsky, & Reed, 2010; Stahlman et al., 2015). Exposure to these negative consequences of disclosure may create a hostile and stressful social environment, thereby worsening mental and physical health (Meyer, 2003; Rodriguez-Hart et al., 2017).

In sub-Saharan Africa, two of the most commonly reported manifestations of stigma related to same-sex sexual practices are (1) enacted or experienced stigma, which refers to behavioral expressions of stigma such as physical violence, blackmail, and denial of healthcare services and (2) perceived or anticipated stigma, which refers to a fear or expectation that enacted stigma may occur, such as fear of seeking health care (Baral et al., 2011; Henry, Awondo, Fugon, Yomb, & Spire, 2012; King et al., 2013; Risher et al., 2013; Sekoni, Ayoola, & Somefun, 2015). In 2014, legislation passed by the Nigerian government recommended harsh punishments for MSM and TGW and persons perceived to be promoting homosexuality. Following the passage of this law, MSM and TGW reported increased fear and avoidance of healthcare services (Schwartz et al., 2015). In 2017, Nigerian police raided an event organized to promote HIV testing in Lagos and arrested men who were accused of engaging in same-sex sexual practices, a crime punishable by up to 14 years in prison (British Broadcasting Corporation, 2017). Prior research has shown that blackmail and beatings due to same-sex sexual practices are commonly experienced by Nigerian MSM and TGW and are linked to increased risk of genitourinary diseases (Stromdahl et al., 2019). This could be partially explained by stigma leading to higher risk sexual behaviors, such as increased condomless sex (Coulaud et al., 2020; Stahlman et al., 2016) and decreased engagement with healthcare systems (Mason et al., 2015). We have previously proposed a model whereby disclosure of same-sex sexual practices can lead to increased hierarchical classes of stigma, then suicidal ideation, then condomless sex with casual sex partners, and finally increased risk of HIV and other STIs (Rodriguez-Hart et al., 2017).

Understanding disclosure patterns of same-sex sexual practices among MSM and TGW communities in sub-Saharan Africa is therefore critical because these communities are marginalized, stigmatized, and have an exceptionally high burden of HIV and other STIs. Nigeria is the most populated country in sub-Saharan Africa, and its residents account for almost one out of every ten people living with HIV around the world and about 10% of new diagnoses annually (UNAIDS, 2014, 2015). In some communities of Nigerian MSM and TGW, HIV prevalence as high as 44–66% has been reported (Keshinro et al., 2016). The disproportionate burden of HIV among MSM and TGW is driven partially by physiologic factors such as the increased transmissibility of HIV via condomless anal as compared to vaginal sex and partially by social and cultural factors such as the suboptimal quality and coverage of preventive healthcare services available to MSM and TGW (Beyrer et al., 2012, 2013; Rodriguez-Hart et al., 2017).

A structural approach to managing the stigma associated with same-sex sexual practices, including any stigma resulting from disclosure thereof, is necessary to optimize quality of care (Djomand, Quaye, & Sullivan, 2014). In Swaziland, a structural stigma-reduction intervention package has been implemented that encourages disclosure as a means of increasing uptake of evidence-based and rights-affirming care for MSM and TGW that includes couples’ counseling, provision of condoms and condom-compatible lubricants, anal cancer screening, and laboratory-based STI diagnosis (Risher et al., 2013). While the Nigerian healthcare system is stratified into tiers operated at the local, state, and federal levels with inconsistent harmonization (Akhtar, 1991), there is increasing recognition at some levels of the importance of engaging MSM and TGW to curb the spread of HIV (National Agency for the Control of AIDS, 2019). Support in Nigeria is shifting toward nongovernmental organizations that deliver health care in “safe spaces” like TRUST/RV368 to address specific needs of MSM and TGW (Charurat et al., 2015; Crowell et al., 2019; Emmanuel et al., 2019; Ibiloye, Decroo, Eyona, Eze, & Agada, 2018; Nowak et al., 2019; Tun et al., 2018).

In these analyses, we separated disclosure out from previously explored stigma classes to identify the proportion of participants who disclosed their same-sex sexual practices and evaluate whether disclosure could be an avenue for intervention to promote engagement with MSM- and TGW-friendly clinics. We also further explored the relationships between disclosure to a family member and/or HCP with condom use, stigma, and discrimination.

## Method

### Participants

Participants in the TRUST/RV368 cohort study were Nigerian MSM and TGW recruited at trusted community health centers in Abuja and Lagos that provide HIV/STI-focused healthcare services. These facilities were developed by local nongovernmental organizations with support from the President’s Emergency Plan for AIDS Relief (PEPFAR) to provide prevention and treatment services. Staff were trained in MSM and TGW healthcare needs and provided care in facilities that afforded safe spaces for MSM and TGW to socialize. Healthcare visits included education about HIV and STI prevention and treatment, including the use of condoms and water-based lubricants that were freely available throughout the clinical and nonclinical spaces in each facility.

Recruitment was conducted using respondent-driven sampling (RDS) in order to facilitate enrollment of hard-to-reach populations, as previously described (Baral et al., 2015; Charurat et al., 2015). Briefly, recruitment began with identification of several initial volunteers, called “seeds,” who were identified through local nongovernmental organizations and key opinion leaders and selected to represent a variety of ages, socioeconomic classes, and neighborhoods in each city where the study was conducted. Each “seed” participant was given three coupons to provide to other MSM and TGW in his or her social network. Each enrolled participant then received three more coupons to distribute. Incentives were provided for participation in study visits (Naira 2000–3400, equal to about US$6–11, depending on visit) and for referrals (Naira 1500, equal to about US$5). Eligible participants for this study had to present with a valid RDS coupon, have been assigned male gender at birth, be over 16 years old in Abuja or over 18 years old in Lagos (reflecting differences in local IRB guidance), report receptive or insertive anal sex in the 12 months prior to enrollment, and speak English or Hausa. Nigeria has over 500 native languages with English as the official language used widely in the media and increasingly as a first language in Nigerian urban centers, while Hausa is the second most commonly spoken language (Blench, 2019).

Participants who enrolled in the cohort between March 2013 and March 2018 and answered both baseline questions about disclosure to a family member and/or a HCP were included in these analyses. All analyses were cross-sectional using data from the time of enrollment.

### Measures

Upon enrollment, data on participant demographics and sexual behaviors were collected by a trained interviewer using a standardized questionnaire. A study physician performed a medical examination and recorded each participant’s medical history. Participants underwent counseling and testing for HIV and other STIs. HIV status was determined using a parallel algorithm of rapid tests with Determine^®^ (Alere, Waltham, MA) and Uni-Gold^®^ (Trinity Biotech, Wicklow, Ireland) kits and a third tie-breaker with HIV-1/2 Stat-Pak (Chembio Diagnostics, Medford, NY), if needed. All testing was performed according to package inserts. Enrollment evaluations were scheduled across two study visits approximately two weeks apart.

Participants were categorized based on their responses to two questions: (1) “Have you disclosed to any member of your family that you have sex with other men or that you are attracted to other men?” and (2) “Have you disclosed to any healthcare worker that you have sex with other men or that you are attracted to other men?” Age, gender identity, sexual orientation, education, occupation, and marital status were each assessed by self-report. Participants were asked to describe the frequency of condom use during insertive anal and receptive anal sex using the categories “always,” “almost always,” “half the time,” “almost never,” and “never.” They were also asked whether they had ever experienced a variety of potential indicators of stigma and discrimination, such as fear of accessing healthcare services, avoidance of health care, and refusal of health care due to their MSM and TGW status. These indicators reflect types of perceived and experienced stigma related to same-sex sexual practices that have been previously observed in Western and Southern Africa (Rodriguez-Hart et al., 2018; Stahlman et al., 2016).

Data from clinical evaluations and participant interviews were collected on paper case report forms, imported into an electronic database using TeleForm (Hewlett-Packard Inc., Palo Alto, CA), and verified for accuracy by a dedicated staff member.

### Statistical Analyses

Separate analyses were conducted to compare groups reporting disclosure of same-sex sexual practices to a family member and/or a HCP. Demographic characteristics, condom use, and experiences of stigma were compared between these groups using Pearson’s chi-squared test or Fisher’s exact test for categorical variables and *t* test for continuous variables. In separate models for disclosure to a family member and to a HCP, Poisson regression with robust error variance was used to estimate the relative risk (RR) of disclosure and 95% confidence intervals (CIs) associated with pre-specified factors of interest such as age, gender identity, sexual orientation, education, marital status, city, and enrollment year (Zou, 2004). Multivariable models included all pre-specified factors of interest. A two-sided type I error less than 5% was considered statistically significant. To help evaluate separate effects of disclosure to either a family member or HCP as well as the impact of overlapping disclosure to both a family member and HCP, comparisons were repeated across all four possible combinations of disclosure: disclosure to neither a family member nor HCP, disclosure to a family member only, disclosure to a HCP only, or disclosure to both a family member and HCP. A single multinomial logistic regression model was used to generate adjusted RRs and 95% CIs for factors associated with each of these categories as compared to the nondisclosing reference group. For all analyses, missing data were carried backward from the scheduled 3-month follow-up study visit, if available, or were categorized as unknown. Analyses were performed using Stata 14.2 (StataCorp LP, College Station, TX).

## Results

### Demographics and Disclosure of Same-Sex Sexual Practices

A total of 2591 participants were enrolled between March 2013 and May 2019, including 34 who did not answer questions about disclosure of same-sex sexual practices and were excluded from these analyses. Of the remaining 2557 participants included in these analyses, 1885 (73.7%) were from Abuja and 672 (26.3%) from Lagos. The median age of participants was 23 years with interquartile range (IQR) 21–27 years. Same-sex sexual practice disclosure among participants was generally uncommon, with only 925 (36.2%) reporting some sort of disclosure, including 192 (7.5%) who had disclosed only to a family member, 541 (21.2%) only to a HCP, and 192 (7.5%) to both a family member and HCP. Demographic and other characteristics of the study population, stratified by disclosure of same-sex sexual practices to a family member and/or HCP, are shown in Table 1 and Supplemental Table 1.

**Table 1:** Characteristics of Nigerian men who have sex with men who reported on disclosure of same-sex sexual practices to a family member and/or a healthcare provider

Characteristic	Disclosed to a family member		*χ*^2^ (*p*)	Disclosed to a healthcare provider		*χ*^2^ (*p*)
	No (*N* = 2173)	Yes (*N* = 384)		No (*N* = 1824)	Yes (*N* = 733)	
Age						
< 22 years	726 (33.4)	117 (30.5)	2.46 (.29)	665 (36.5)	178 (24.3)	**39.13 (< .01)**
22–30 years	1226 (56.4)	233 (60.7)		1000 (54.8)	459 (62.6)	
> 30 years	221 (10.2)	34 (8.9)		159 (8.7)	96 (13.1)	
Gender identity						
Cisgender man	1758 (80.9)	284 (74.0)	**10.74 (< .01)**	1480 (81.5)	562 (76.7)	**7.10 (.03)**
Transgender woman	212 (9.8)	56 (14.6)		183 (10.0)	85 (11.6)	
Other/unknown	203 (9.3)	44 (11.5)		161 (8.8)	86 (11.7)	
Sexual orientation						
Gay/homosexual	666 (30.6)	156 (40.6)	**15.33 (< .01)**	579 (31.7)	243 (33.2)	**26.67 (< .01)**
Bisexual	1489 (68.5)	224 (58.3)		1240 (68.0)	473 (64.5)	
Other/unknown	18 (0.8)	4 (1.0)		5 (0.3)	17 (2.3)	
Education level						
Junior secondary or less	316 (14.5)	21 (5.5)	**32.07 (< .01)**	285 (15.6)	52 (7.1)	**73.27 (< .01)**
Senior secondary	1145 (52.7)	195 (50.8)		987 (54.1)	353 (48.2)	
Higher than senior secondary	692 (31.8)	163 (42.4)		543 (29.8)	312 (42.6)	
Unknown	20 (0.9)	5 (1.3)		9 (0.5)	16 (2.2)	
Marital status						
Single/never married	1947 (89.6)	352 (91.7)	7.57 (.06)	1644 (90.1)	655 (89.4)	**25.69 (< .01)**
Married/living with a woman	137 (6.3)	15 (3.9)		124 (6.8)	28 (3.8)	
Living with a man	21 (1.0)	8 (2.1)		16 (0.9)	13 (1.8)	
Divorced/widowed/other	68 (3.1)	9 (2.3)		40 (2.2)	37 (5.0)	
Site						
Abuja	1655 (76.2)	230 (59.9)	**44.57 (< .01)**	1454 (79.7)	431 (58.8)	**118.06 (< .01)**
Lagos	518 (23.8)	154 (40.1)		370 (20.3)	302 (41.2)	
HIV status						
Uninfected	1053 (48.5)	141 (36.7)	**28.05 (< .01)**	925 (50.7)	269 (36.7)	**68.45 (< .01)**
Living with HIV	747 (34.4)	196 (51.0)		557 (30.5)	386 (52.7)	
Unknown	373 (17.2)	47 (12.2)		342 (18.8)	78 (10.6)	
Enrollment year						
2013	463 (21.3)	73 (19.0)	**39.97 (< .01)**	430 (23.6)	106 (14.5)	**117.94 (< .01)**
2014	427 (19.7)	70 (18.2)		328 (18.0)	169 (23.1)	
2015	318 (14.6)	97 (25.3)		246 (13.5)	169 (23.1)	
2016	240 (11.0)	54 (14.1)		181 (9.9)	113 (15.4)	
2017	297 (13.7)	46 (12.0)		236 (12.9)	107 (14.6)	
2018	342 (15.7)	32 (8.3)		322 (17.7)	52 (7.1)	
2019	86 (4.0)	12 (3.1)		81 (4.4)	17 (2.3)	

All data are presented as n (column percentage). *p* values were calculated using Pearson’s chi-squared (*χ*^2^) test. Comparisons were repeated using Fisher’s exact test for variables with any cell count ≤ 5 and yielded similar, statistically significant results. Statistically significant *χ*^2^ and *p* values are shown in bold

### Factors Associated with Disclosure of Same-Sex Sexual Practices

In unadjusted analyses, disclosure of same-sex sexual practices to a family member was more common among transgender women, more highly educated participants, participants living with HIV or with unknown HIV status, participants in Lagos, and during specific years of enrollment (Table 2). Participants who self-identified as bisexual and were married or living with a woman were less likely to disclose to family. After adjusting for all evaluated factors, higher education level, recruitment at the Lagos clinical care site, and living with HIV were each independently associated with an increased likelihood of disclosure to family. As compared to participants with gay/homosexual/other sexual orientation, those reporting a bisexual orientation were less likely to disclose same-sex sexual practices to a family member.

**Table 2:** Unadjusted and adjusted analyses of factors associated with disclosure of same-sex sexual practices

	Disclosed to a family member Relative risk (95% CI)		Disclosed to a healthcare provider Relative risk (95% CI)	
	Unadjusted	Adjusted	Unadjusted	Adjusted
Age				
< 22 years	Reference	**–**	**–**	**–**
22–30 years	1.02 (0.99–1.04)	1.01 (0.98–1.04)	**1.08 (1.05**–**1.12)**	**1.06 (1.03**–**1.10)**
> 30 years	1.00 (0.95–1.04)	0.99 (0.95–1.04)	**1.14 (1.08**–**1.19)**	**1.12 (1.07**–**1.18)**
Gender identity				
Cisgender man	Reference	**–**	**–**	**–**
Transgender woman	**1.06 (1.02**–**1.11)**	1.04 (1.00–1.09)	1.03 (0.99–1.08)	1.01 (0.96–1.05)
Other/unknown	1.03 (0.99–1.08)	1.03 (0.99–1.08)	**1.06 (1.01**–**1.11)**	**1.05 (1.00**–**1.10)**
Sexual orientation				
Gay/homosexual/other/unknown	Reference	**–**	**–**	**–**
Bisexual	**0.95 (0.92**–**0.98)**	**0.96 (0.93**–**0.98)**	0.98 (0.95–1.00)	0.98 (0.96–1.01)
Education level				
Junior secondary or less/unknown	Reference	**–**	**–**	**–**
Senior secondary	**1.07 (1.04**–**1.10)**	**1.05 (1.01**–**1.08)**	**1.06 (1.02**–**1.10)**	1.01 (0.97–1.05)
Higher than senior secondary	**1.11 (1.07**–**1.15)**	**1.10 (1.06**–**1.14)**	**1.15 (1.10**–**1.20)**	**1.08 (1.03**–**1.13)**
Marital status				
Single/never married	Reference	**–**	**–**	**–**
Married/living with a woman	**0.95 (0.91**–**1.00)**	0.99 (0.94–1.03)	**0.92 (0.87**–**0.97)**	**0.92 (0.87**–**0.97)**
Living with a man	1.11 (0.97–1.26)	1.05 (0.92–1.19)	1.13 (0.99–1.28)	1.01 (0.89–1.14)
Divorced/widowed/other	0.97 (0.91–1.03)	0.97 (0.91–1.04)	**1.15 (1.07**–**1.24)**	**1.13 (1.04**–**1.22)**
Site				
Abuja	Reference	**–**	**–**	**–**
Lagos	**1.10 (1.06**–**1.13)**	**1.11 (1.05**–**1.16)**	**1.18 (1.14**–**1.22)**	**1.22 (1.16**–**1.29)**
HIV status				
Uninfected	Reference	**–**	**–**	**–**
Living with HIV	**1.07 (1.03**–**1.10)**	**1.05 (1.02**–**1.08)**	**1.13 (1.09**–**1.17)**	**1.09 (1.05**–**1.13)**
Unknown	**1.07 (1.04**–**1.10)**	0.98 (0.94–1.02)	**1.13 (1.09**–**1.16)**	0.96 (0.92–1.01)
Enrollment year				
2013	Reference	**–**	**–**	**–**
2014	1.00 (0.97–1.04)	**0.96 (0.92**–**1.00)**	**1.12 (1.07**–**1.17)**	1.03 (0.98–1.08)
2015	**1.08 (1.04**–**1.13)**	1.00 (0.96–1.06)	**1.17 (1.12**–**1.23)**	1.03 (0.98–1.09)
2016	1.04 (1.00–1.09)	0.97 (0.92–1.02)	**1.16 (1.10**–**1.21)**	1.01 (0.96–1.07)
2017	1.00 (0.96–1.04)	0.97 (0.93–1.02)	**1.10 (1.04**–**1.15)**	1.04 (0.99–1.09)
2018	**0.96 (0.92**–**0.99)**	**0.94 (0.90**–**0.98)**	**0.95 (0.91**–**0.99)**	**0.92 (0.88**–**0.97)**
2019	0.99 (0.93–1.05)	0.97 (0.91–1.03)	0.98 (0.91–1.05)	0.96 (0.89–1.03)

Unadjusted and adjusted Poisson regression models with robust error variance were used to calculate relative risk and 95% confidence intervals for factors associated with same-sex sexual practice disclosure to family and HCP. Because of small cell sizes, the “other/unknown” and “unknown” values for sexual orientation and education level, respectively, were collapsed into the reference group in each model. Adjusted models included all factors listed in the table. Statistically significant associations are shown in bold

In unadjusted analyses, disclosure of same-sex sexual practices to a HCP was more common among older participants, those with other/unknown gender identity, more highly educated participants, participants living with HIV or with unknown HIV status, participants in Lagos, and during specific years of enrollment (Table 2). After adjusting for other factors, older age, other/unknown gender identity, higher education level, recruitment at the Lagos clinical care site, and living with HIV were each independently associated with an increased likelihood of disclosure to family. Participants who were married or living with a woman were less likely to disclose to a HCP in both the unadjusted and adjusted models.

The multinomial logistic regression model revealed similar trends (Supplemental Table 2). Older age was associated with greater likelihood of membership in one of the two groups that disclosed to a HCP. Factors associated with increased likelihood of all three combinations of disclosure as compared to nondisclosure included higher than senior secondary education (as compared to junior secondary or less), recruitment at the Lagos site (as compared to Abuja), and living with HIV (as compared to HIV-uninfected).

### Sexual Behaviors and Condom Use

**Fig. 1:**
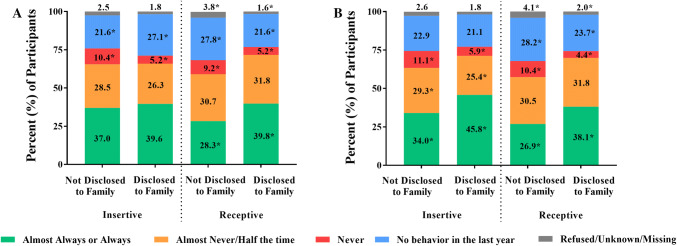
Self-reported condom use during insertive and receptive anal sex, stratified by disclosure of same-sex sexual practices to **a** family and **b** healthcare providers. Pearson’s chi-squared test was used to compare the proportion of participants reporting each frequency of condom use stratified by disclosure status. Condom use during insertive anal sex and receptive anal sex was considered separately. Statistically significant differences between groups are represented by an asterisk (*)

Participants who disclosed their same-sex sexual practices to a family member had a slightly younger age of coitarche with male partners, which occurred at a mean age of 16.5 years (SD, 4.0), compared to 17.3 (SD, 4.6) years among those who had not disclosed to family (*t *= 3.06, *p *< . 01). No significant difference was observed in age of coitarche with female partners between participants who had and had not disclosed their same-sex sexual practices to family (17.2 [SD, 4.3] vs. 17.1 [SD, 4.2] years, *t *= −.47, *p *= .64). Compared to participants who had not disclosed their same-sex sexual practices to a family member, participants who had disclosed were more likely to report always or almost always using condoms during receptive but not insertive anal sex (Fig. 1a).

Participants who disclosed their same-sex sexual practices to a HCP had a slightly older age of coitarche with male partners, which occurred at a mean age of 17.6 (SD, 4.7) years compared to 17.0 (SD, 4.5) years among those who had not disclosed to a HCP (*t *= −2.96, *p *< .01). No significant difference was observed in age of coitarche with female partners between participants who had and had not disclosed their same-sex sexual practices to a HCP (17.3 [SD, 4.4] vs. 17.0 [SD, 4.1] years, *t *= −1.22, *p *= .22). Compared to participants who had not disclosed their same-sex sexual practices to a HCP, participants who had disclosed were more likely to report always or almost always using condoms during both insertive and receptive anal sex (Fig. 1b).

Similar inferences were drawn from comparisons across groups defined by all four possible patterns of disclosure (Supplemental Fig. 1). Participants who disclosed same-sex sexual practices to a family member only were more likely to report condom use during receptive but not insertive anal sex. Participants who disclosed same-sex sexual practices to a HCP, with or without concurrent disclosure to a family member, were more likely to report condom use during both insertive and receptive anal sex as compared to participants who did not report any disclosure.

### Experiences of Stigma

**Fig. 2:**
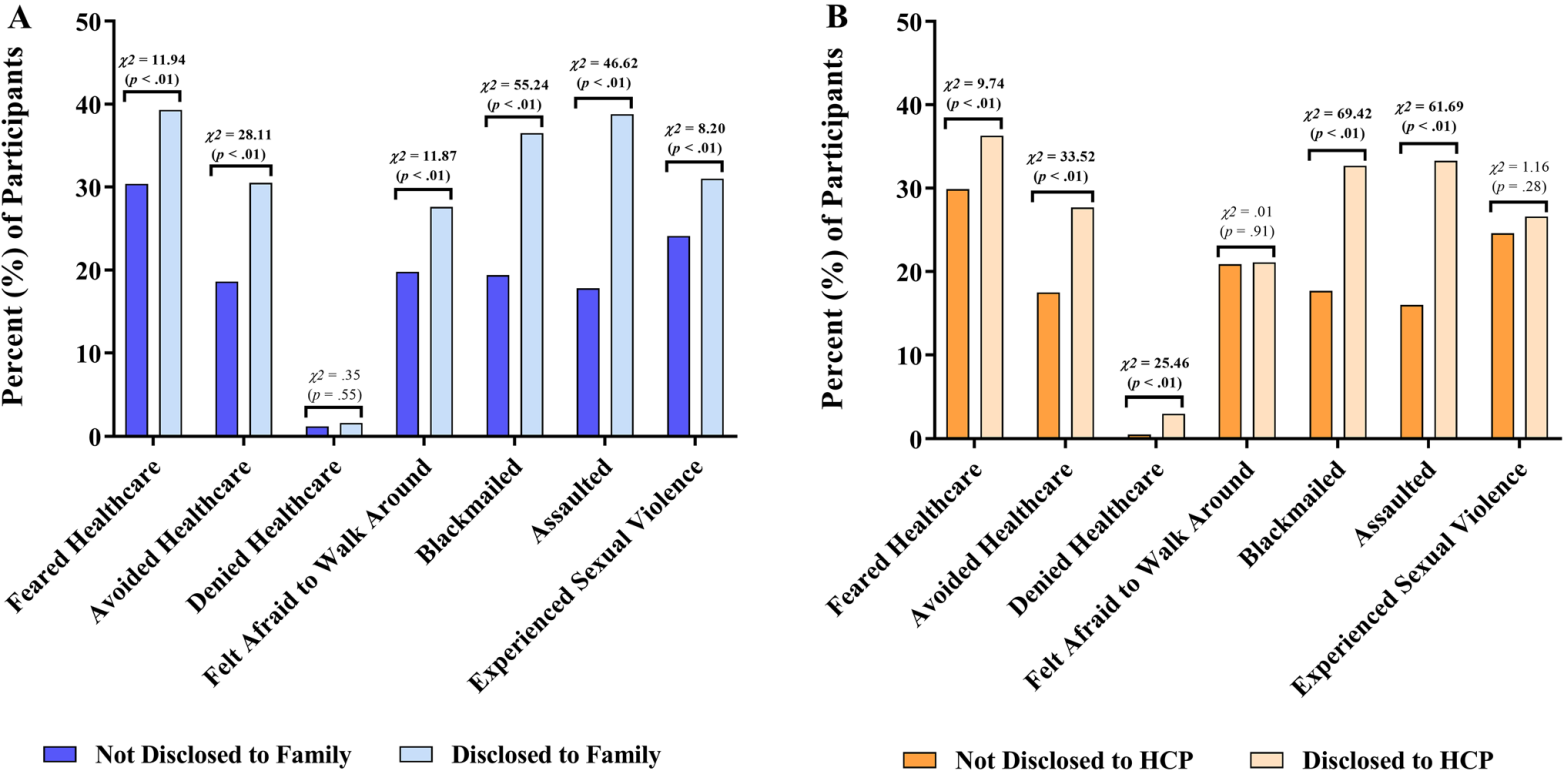
Indicators of perceived and experienced stigma due to same-sex sexual practices, stratified by disclosure of same-sex sexual practices to **a** family and **b** healthcare providers. Bar height represents the percentage of study participants who reported each indicator of stigma upon enrollment. Pearson’s chi-squared test was used to compare the proportion of participants reporting each stigma indicator, stratified by disclosure status. Statistically significant differences between groups are shown in bold

Compared to those who had not disclosed to a family member, participants who had disclosed their same-sex sexual practices to a family member were more likely to report fear of seeking health services (39.3% vs. 30.4%, *χ*^2^ = 11.94, *p *< .01) and avoidance of healthcare services (30.5% vs. 18.6%, *χ*^2^ = 28.11, *p *< . 01) but were no more likely to be denied healthcare services (1.6% vs. 1.2%, *χ*^2^ = .35, *p *= .55) because they were MSM and TGW. Furthermore, those who had disclosed same-sex sexual practices to a family member were more likely to have felt afraid to walk around (27.6% vs. 19.8%, *χ*^2^ = 11.87, *p *< .01), experienced blackmail (36.5% vs. 19.4%, *χ*^2^ = 55.24, *p *< .01), and experienced assault (38.8% vs. 17.8%, *χ*^2^ = 46.62, *p *< .01) because they were MSM and TGW, when compared to those who had not made a similar disclosure (Fig. 2a).

Similarly, compared to those who had not disclosed to a HCP, participants who had disclosed their same-sex sexual practices to a HCP were more likely to report fear of seeking health services (36.3% vs. 29.9%, *χ*^2^ = 9.74, *p * <  .01) and avoidance of healthcare services (27.7% vs. 17.5%, *χ*^2^ = 33.52, *p *< .01). Unlike disclosure to a family member, disclosure to a HCP was associated with a significantly increased risk of being denied healthcare services (3.0% vs. .5%, *χ*^2^ = 25.46, *p *< .01). Participants who had disclosed same-sex sexual practices to a HCP were also more likely to have experienced both blackmail (32.7% vs. 17.7%, *χ*^2^ = 69.42, *p *< .01) and assault (33.3% vs. 16.0%, *χ*^2^ = 61.69, *p *< .01) because they were MSM and TGW, when compared to those who had not made a similar disclosure (Fig. 2b).

Similar inferences were drawn from comparisons across groups defined by all four possible patterns of disclosure (Supplemental Fig. 2). Disclosure to a family member was not associated with denial of health care, but denial of health care was more common in both groups that had disclosed to a HCP. A similar pattern was observed with sexual violence. Disclosure to a HCP was not associated with fear of walking around, but more participants in the groups who disclosed to a family member reported this fear. Other stigma indicators were generally increased among participants who had made any disclosure(s) as compared to the nondisclosure group.

## Discussion

Disclosure of same-sex sexual practices to a family member or HCP were each relatively uncommon among Nigerian MSM and TGW in this study. In each case, disclosure was associated with a larger proportion of participants reporting condom use, consistent with results from prior studies suggesting that disclosure enhances social support, improves quality of health care, and improves treatment outcomes in MSM and TGW populations (Schwartz et al., 2015; Stahlman et al., 2015). However, these apparent beneficial effects were countered by strong relationships between disclosure and stigma, which may have discouraged utilization of healthcare services.

Disclosure of same-sex sexual practices among Nigerian MSM and TGW in this study was generally less common than has been observed in settings with less criminalization and stigmatization of homosexuality. The disclosure rates of about 15% and 29% to a family member and a HCP, respectively, among MSM and TGW in this study were substantially lower than the rates of about 75% and 56%, respectively, which have been reported from South Africa, where MSM-friendly legislation exists (Daniels et al., 2018). In contrast, research from countries with laws that criminalize same-sex sexual practices among men generally shows much lower rates of disclosure, including disclosure to a family member by 46% of MSM and TGW in Cameroon, to HCP by 31% of MSM and TGW in Swaziland, and disclosure to a HCP by only 17% of MSM and TGW participants in a large cross-sectional study in Malawi, Namibia, and Botswana (Brown et al., 2016; Fay et al., 2011; Henry et al., 2012). In addition to criminalization, cultural and societal factors may also contribute to disclosure practices and should be considered when designing affirmative and targeted population-based HIV and STI prevention intervention. In China, a country without anti-homosexuality laws but with extralegal consequences for same-sex sexual practices, the overall disclosure rate to HCP was found to be only 16% (Tang et al., 2017). In the African context, cultural stigmatization has created barriers to MSM- and TGW-focused healthcare services that are compounded by criminalizing legislation that creates fear of arrest and brutality (Beyrer, 2014; Essien & Aderinto, 2009; Semugoma, Nemande, & Baral, 2012).

Stigma was associated with disclosure of same-sex sexual practices to both family and HCP in this study. Participants in this study reported both perceived and experienced stigma because they were MSM and TGW, including specific stigma-related barriers to healthcare engagement. Notably, although denial of health care was generally uncommon, MSM and TGW who disclosed their same-sex sexual practices to HCP were substantially more likely to be denied health care. The stigma and discrimination experienced by Nigerian MSM and TGW are in some ways legitimized by Nigeria’s anti-homosexuality legislation, which has resulted in increased stigma that acts as a barrier to disclosure of sexual practices (Schwartz et al., 2015). Similar to the observation in this study, same-sex practice disclosure among MSM and TGW in Swaziland was found to be associated with stigma and fear of seeking healthcare services (Risher et al., 2013). In Gambia, studies show several associations between disclosure of same-sex sexual practices by MSM and human rights abuses such as physical assaults, arbitrary arrests, and threats of decapitation. While important, decriminalization of homosexuality does not eliminate social and cultural sources of stigma. As the first and only African nation to legalize unions between same-sex couples, South Africa is one of sub-Saharan Africa’s most legislatively accepting countries (Pew Research Center, 2019). However, MSM and TGW still experience significant stigma, discrimination, and poor reception from HCP in South Africa (Rispel, Metcalf, Cloete, Moorman, & Reddy, 2011). A study that evaluated the impact of decriminalizing laws in South Africa, Botswana, Malawi, and Namibia found that human rights abuses such as police harassment and denial of housing persisted despite legislative changes but blackmail and fear were diminished (Zahn et al., 2016). Legal and social change must occur in tandem in order to optimally promote community acceptance, decrease stigma, and encourage disclosure of same-sex sexual practices to family and HCP. This could possibly improve healthcare engagement and access to appropriate preventive health services.

In this study, certain key factors were found to be associated with disclosure of same-sex sexual practices among MSM and TGW. Increased educational level was strongly associated with disclosure to both family and HCP, underscoring the general importance of education possibly facilitating disclosure. The reasons for disclosure being more common among participants living in Lagos are uncertain. Both Lagos and Abuja are large urban centers with reports of discrimination and human rights abuses affecting MSM and TGW (British Broadcasting Corporation, 2017; Sekoni et al., 2015; Stromdahl et al., 2019). Understanding the social, behavioral, and environmental factors that positively influenced disclosure in Lagos could inform HIV prevention approaches.

Same-sex practice disclosure to either a family member or HCP was associated with more condom use. A prior study from China similarly found that nondisclosure was associated with riskier same-sex sexual practices among gay and bisexual men (Zhao et al., 2016). Despite the potential positive relationship between disclosure and condom uptake, same-sex sexual practice disclosure was also associated with increased perceived and experienced stigma. Prior data suggest that knowledge and attitudes of HCP are key barriers to disclosure of same-sex sexual practices (Ruben & Fullerton, 2018). Training HCP to demonstrate awareness through a patient management algorithm that incorporates proactive inquiry about sexual practices could reduce fear of disclosure and improve care and treatment outcomes. Tailoring interventions to the needs of individuals and communities who are less likely to disclose their same-sex sexual practices may improve results. National recognition and support of nongovernmental organizations that provide community-engaged care to MSM and TGW in safe spaces may help to optimize the gains from disclosure while minimizing stigma.

There were a number of strengths and limitations of these analyses. The respondent-driven sampling technique enabled the enrollment of a highly marginalized and hard-to-reach population of MSM and TGW. The use of standardized questionnaires enabled the collection of detailed information regarding study population demographics, same-sex practice disclosure, and associated stigma. However, potentially sensitive and stigmatizing data collected via self-report may suffer from a number of biases, including response and recall biases. The population included in these analyses was recruited in two large urban centers in Nigeria and may not be representative of populations in other areas. Recruitment at trusted community health centers specially staffed to create safe spaces for MSM and TGW may have biased the study population toward a greater likelihood of disclosure, although this bias was mitigated by the respondent-driven sampling methodology that reached participants who had not previously accessed these facilities. Only individuals who could provide informed consent in English or Hausa were eligible for this study, potentially biasing the study population toward inclusion of individuals with specific educational, geographic, or ethnic backgrounds associated with these languages. There was some overlap of disclosure groups, potentially confounding interpretation of the individual effects of disclosure to either a family member or a HCP; however, almost all inferences were robust to sensitivity analyses that re-categorized participants into four mutually exclusive groups representing all possible patterns of disclosure to a family member and/or a HCP. The primary dichotomous analyses were retained to enable easier visualization and interpretation of the relationships that were confirmed by the sensitivity analyses. While we have provided theoretical pathways through which the factors evaluated may be associated with disclosure of same-sex sexual practices, the cross-sectional nature of these analyses limits our ability to infer any causal relationships. These analyses did not distinguish between voluntary and involuntary disclosure of same-sex sexual practices.

This study revealed a complicated relationship between disclosure and same-sex sexual practices by Nigerian MSM and TGW. Cultural and behavioral implications of disclosure must be considered in designing interventions to engage MSM and TGW in healthcare and HIV prevention services. This study corroborates prior work indicating that interventions to encourage disclosure must be accompanied by larger campaigns to educate communities, create safe spaces to access health care, decrease stigmatization, and promote policies of acceptance. Improved disclosure practices within safe spaces may enhance engagement of MSM and TGW in healthcare and HIV prevention services.

## Electronic Supplementary Material

Below is the link to the electronic supplementary material.

Supplementary material 1 (PDF 434 kb)
