# Supplementary material for: Disclosure of Same-Sex Sexual Practices to Family and Healthcare Providers by Men Who Have Sex with Men and Transgender Women in Nigeria
Source: Arch Sex Behav. 2020 Mar 19;50(4):1665–76. doi: 10.1007/s10508-020-01644-8 (PMC8017753; doi:10.1007/s10508-020-01644-8)
Supplement: Supplementary file 1 — Supplementary material 1 (PDF 434 kb) [file 10508_2020_1644_MOESM1_ESM.pdf]

SUPPLEMENTAL APPENDIX

Disclosure of Same-sex Practices to Family and Healthcare Providers by Men who have Sex with Men in Nigeria

Table of Contents

Supplemental Table 1. Study Population Characteristics by Mutually-Exclusive Categories of Disclosure 2

Supplemental Table 2. Factors Associated with Disclosure of Same-Sex Practices (Multinomial Model) 3

Supplemental Figure 1. Self-Reported Condom Use during Insertive and Receptive Anal Sex, Stratified by Mutually Exclusive Categories of Disclosure 4

Supplemental Figure 2. Indicators of Perceived and Experienced Stigma due to Same-Sex Practices, Stratified by Mutually Exclusive Categories of Disclosure 5

Supplemental Table 1. Study Population Characteristics by Mutually-Exclusive Categories of Disclosure

Characteristic	Disclosed to Neither a Family Member nor a HCP (N = 1632)	Disclosed to a Family Member Only (N = 192)	Disclosed to a HCP Only (N = 541)	Disclosed to Both a Family and a HCP (N = 192)	*p*-value
**Age**					**<0.001**
< 22 years	593 (36.3)	72 (37.5)	133 (24.6)	45 (23.4)	
22-30 years	889 (54.5)	111 (57.8)	337 (62.3)	122 (63.5)	
> 30 years	150 (9.2)	9 (4.7)	71 (13.1)	25 (13.0)	
**Gender Identity**					**0.015**
Cisgender Man	1335 (81.8)	145 (75.5)	423 (78.2)	139 (72.4)	
Transgender Woman	155 (9.5)	28 (14.6)	57 (10.5)	28 (14.6)	
Other/Unknown	142 (8.7)	19 (9.9)	61 (11.3)	25 (13.0)	
**Sexual Orientation**					**<0.001**
Gay/Homosexual	503 (30.8)	76 (39.6)	163 (30.1)	80 (41.7)	
Bisexual	1125 (68.9)	115 (59.9)	364 (67.3)	109 (56.8)	
Other/Unknown	4 (0.2)	1 (0.5)	14 (2.6)	3 (1.6)	
**Education Level**					**<0.001**
Junior Secondary or Less	268 (16.4)	17 (8.9)	48 (8.9)	4 (2.1)	
Senior Secondary	890 (54.5)	97 (50.5)	255 (47.1)	98 (51.0)	
Higher than Senior Secondary	466 (28.6)	77 (40.1)	226 (41.8)	86 (44.8)	
Unknown	8 (0.5)	1 (0.5)	12 (2.2)	4 (2.1)	
**Marital Status**					**<0.001**
Single/Never Married	1467 (89.9)	177 (92.2)	480 (88.7)	175 (91.1)	
Married/Living with a Woman	115 (7.0)	9 (4.7)	22 (4.1)	6 (3.1)	
Living with a Man	13 (0.8)	3 (1.6)	8 (1.5)	5 (2.6)	
Divorced/Separated/Widowed/Other	37 (2.3)	3 (1.6)	31 (5.7)	6 (3.1)	
**Site**					**<0.001**
Abuja	1330 (81.5)	124 (64.6)	325 (60.1)	106 (55.2)	
Lagos	302 (18.5)	68 (35.4)	216 (39.9)	86 (44.8)	
**HIV Status**					**<0.001**
Uninfected	848 (52.0)	77 (40.1)	205 (37.9)	64 (33.3)	
Living with HIV	472 (28.9)	85 (44.3)	275 (50.8)	111 (57.8)	
Unknown	312 (19.1)	30 (15.6)	61 (11.3)	17 (8.9)	
**Enrollment Year**					**<0.001**
2013	389 (23.8)	41 (21.4)	74 (13.7)	32 (16.7)	
2014	301 (18.4)	27 (14.1)	126 (23.3)	43 (22.4)	
2015	201 (12.3)	45 (23.4)	117 (21.6)	52 (27.1)	
2016	154 (9.4)	27 (14.1)	86 (15.9)	27 (14.1)	
2017	218 (13.4)	18 (9.4)	79 (14.6)	28 (14.6)	
2018	298 (18.3)	24 (12.5)	44 (8.1)	8 (4.2)	
2019	71 (4.4)	10 (5.2)	15 (2.8)	2 (1.0)	

Participants were divided into four mutually-exclusive categories representing all possible patterns of disclosure to a family member and/or a HCP: disclosure to neither a family member nor a HCP, disclosure to a family member only, disclosure to a HCP only, or disclosure to both a family member and a HCP. All data are presented as n (column percentage). Statistical significance was assessed using Pearson's chi-squared test, with significant *p*-values shown in bold.

Supplemental Table 2. Factors Associated with Disclosure of Same-Sex Practices (Multinomial Model)

	Disclosed to a Family Member Only	Disclosed to a HCP Only	Disclosed to Both a Family Member and a HCP
**Age**			
< 22 years	Reference	-	-
22-30 years	0.96 (0.68-1.35)	**1.56 (1.22-2.00)**	**1.70 (1.15-2.51)**
> 30 years	0.48 (0.22-1.03)	**2.07 (1.40-3.04)**	**2.44 (1.36-4.39)**
**Gender Identity**			
Cisgender Man	Reference	-	-
Transgender Woman	1.47 (0.93-2.33)	1.04 (0.73-1.47)	1.37 (0.85-2.20)
Other/Unknown	1.26 (0.75-2.14)	1.35 (0.96-1.90)	**1.67 (1.02-2.73)**
**Sexual Orientation**			
Gay/Homosexual	Reference	-	-
Bisexual	0.74 (0.53-1.02)	0.96 (0.76-1.20)	**0.62 (0.44-0.86)**
**Education Level**			
Junior Secondary or Less	Reference	-	-
Senior Secondary	1.32 (0.75-2.30)	0.90 (0.63-1.27)	**2.70 (1.26-5.81)**
Higher than Senior Secondary	**2.34 (1.31-4.19)**	**1.52 (1.06-2.17)**	**4.68 (2.16-10.13)**
**Marital Status**			
Single/Never Married	Reference	-	-
Married/Living with a Woman	1.10 (0.52-2.33)	**0.59 (0.35-0.98)**	0.48 (0.20-1.17)
Living with a Man	1.18 (0.32-4.31)	0.94 (0.38-2.34)	1.45 (0.49-4.27)
Divorced/Separated/Widowed/Other	0.81 (0.24-2.71)	**2.48 (1.46-4.21)**	1.41 (0.56-3.56)
**Site**			
Abuja	Reference	-	-
Lagos	**2.91 (1.45-5.84)**	**4.21 (2.62-6.75)**	**5.75 (2.56-12.91)**
**HIV Status**			
Uninfected	Reference	-	-
Living with HIV	**1.53 (1.00-2.35)**	**1.74 (1.32-2.30)**	**2.24 (1.44-3.49)**
Unknown	0.85 (0.48-1.53)	0.75 (0.49-1.15)	0.62 (0.29-1.33)
**Enrollment Year**			
2013	Reference	-	-
2014	**0.53 (0.30-0.96)**	1.31 (0.90-1.90)	0.86 (0.48-1.53)
2015	0.99 (0.54-1.81)	1.38 (0.91-2.10)	1.10 (0.60-2.02)
2016	0.80 (0.42-1.52)	1.28 (0.84-1.97)	0.72 (0.38-1.37)
2017	0.62 (0.33-1.16)	1.38 (0.92-2.03)	0.92 (0.52-1.65)
2018	0.66 (0.37-1.18)	**0.63 (0.41-0.98)**	**0.24 (0.10-0.54)**
2019	1.09 (0.51-2.34)	0.98 (0.52-1.85)	0.26 (0.06-1.11)

Multinomial logistic regression was used to calculate relative risk and 95% confidence intervals for factors associated with same-sex practice disclosure to a family member and/or a HCP as compared to a reference group that disclosed to neither a family member nor a HCP. Because of small cell sizes, the "Other/Unknown" and "Unknown" values for sexual orientation and education level, respectively, were collapsed into the reference group for these variables. A single multivariable model included all factors listed in the table. Statistically significant associations are shown in bold.

Supplemental Figure 1. Self-Reported Condom Use during Insertive and Receptive Anal Sex, Stratified by Mutually Exclusive Categories of Disclosure

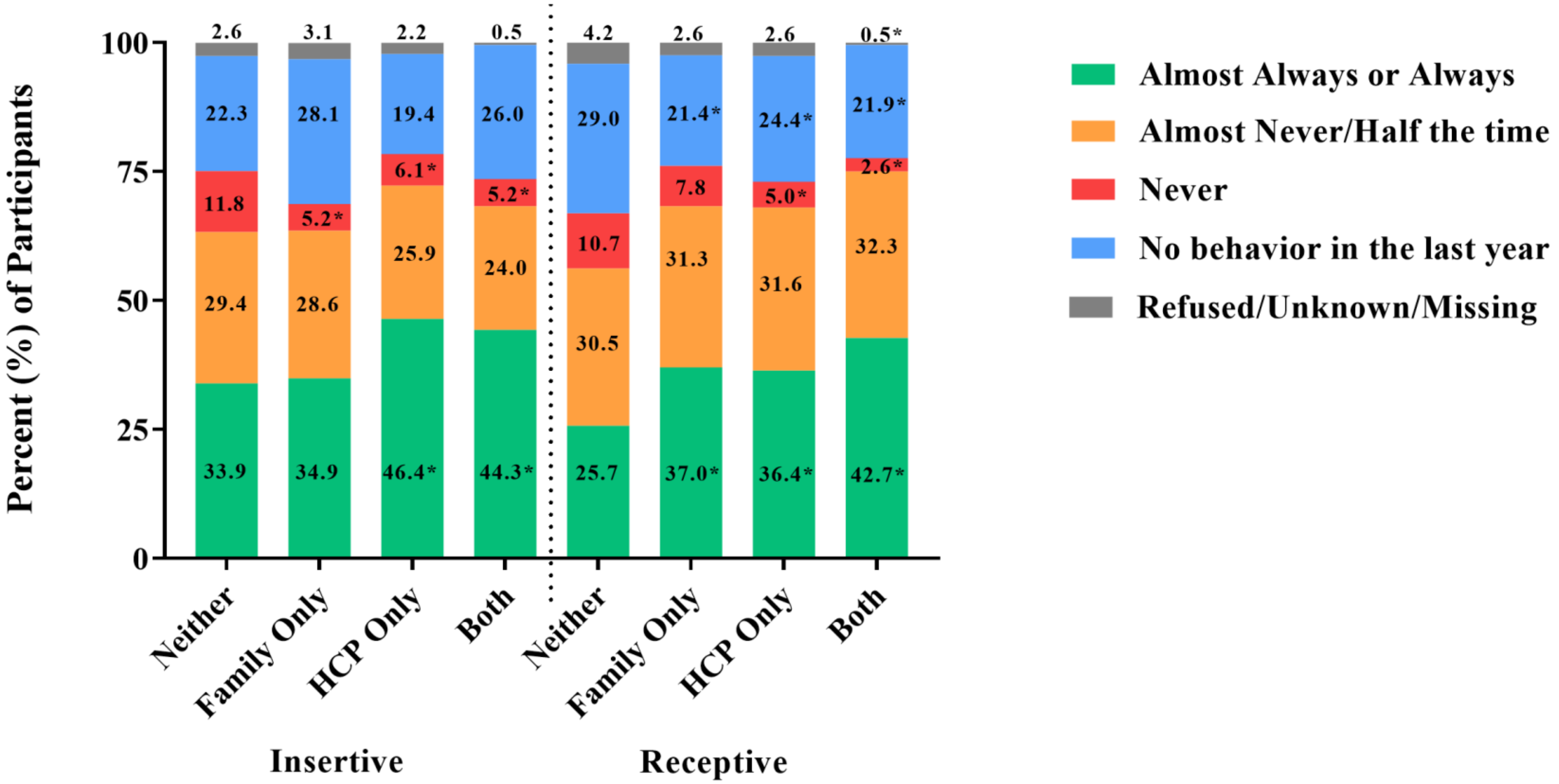

Participants were divided into four mutually-exclusive categories representing all possible patterns of disclosure to a family member and/or a HCP: disclosure to neither a family member nor a HCP, disclosure to a family member only, disclosure to a HCP only, or disclosure to both a family member and a HCP. Pearson's Chi-squared test was used to compare the proportion of participants reporting each frequency of condom use against the group that reported disclosure to neither a family member nor a HCP. Condom use during insertive anal sex and receptive anal sex were considered separately. Statistically significant comparisons are indicated by an asterisk (*).

Supplemental Figure 2. Indicators of Perceived and Experienced Stigma due to Same-Sex Practices, Stratified by Mutually Exclusive Categories of Disclosure

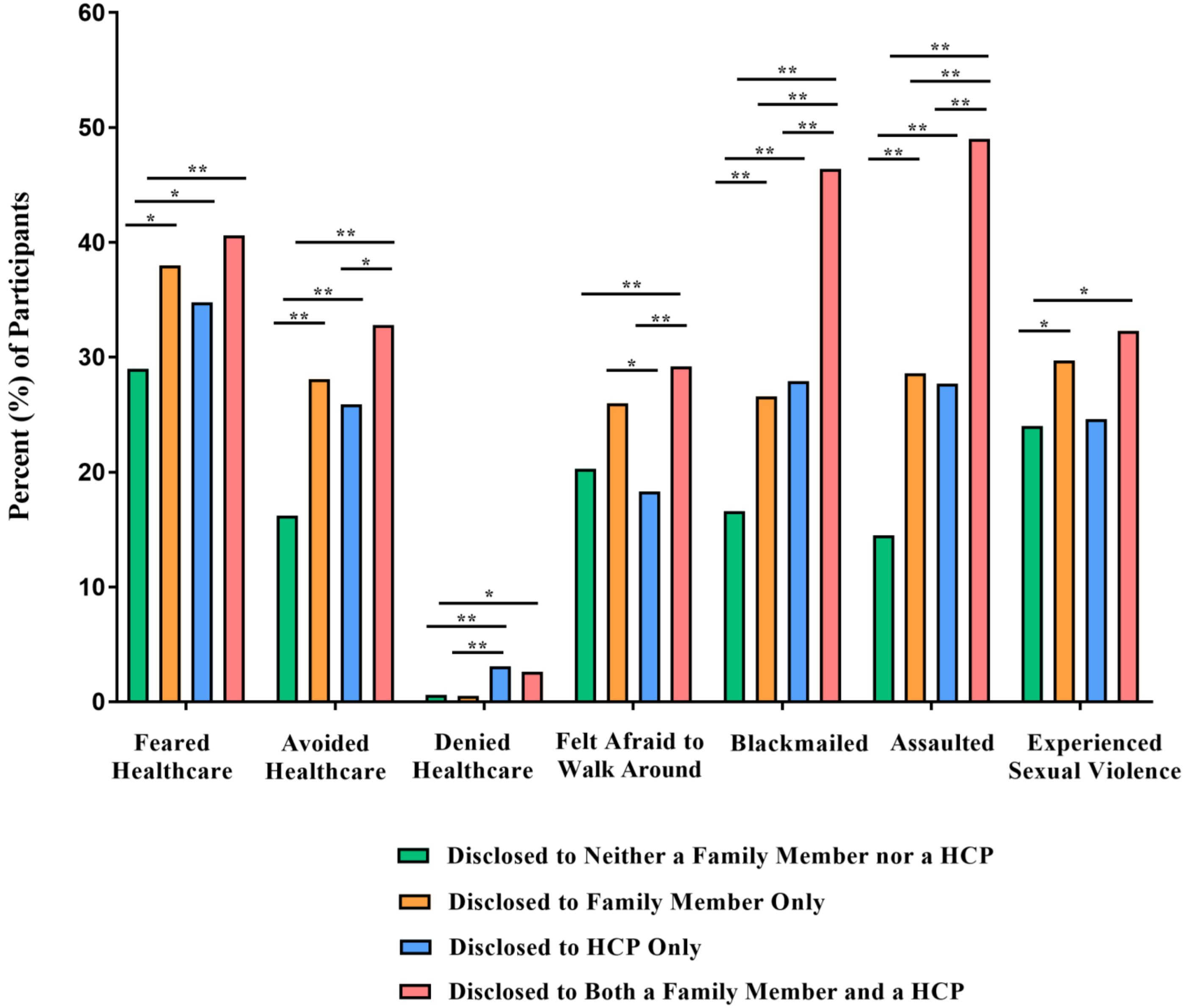

Participants were divided into four mutually-exclusive categories representing all possible patterns of disclosure to a family member and/or a HCP: disclosure to neither a family member nor a HCP, disclosure to a family member only, disclosure to a HCP only, or disclosure to both a family member and a HCP. Bar height represents the percentage of study participants who reported each indicator of stigma upon enrollment. Pearson's Chi-squared test was used to compare the proportion of participants reporting each stigma indicator, stratified by disclosure status. Significant differences between groups are indicated by either one asterisk (*, $p < 0.05$) or two asterisks (**, $p < 0.01$). All other pairwise comparisons were not statistically significant.
